# *N*-{[2,5-Dichloro-4-(1,1,2,3,3,3-hexa­fluoro­prop­oxy)phen­yl]amino­carbon­yl}-2,6-difluoro­benzamide

**DOI:** 10.1107/S1600536808025506

**Published:** 2008-08-13

**Authors:** Yin-hong Liu, Fang-shi Li, Yi Li

**Affiliations:** a Department of Applied Chemistry, College of Science, Nanjing University of Technology, Xinmofan Road No. 5, Nanjing 210009, People’s Republic of China

## Abstract

In the mol­ecule of the title compound, C_17_H_8_Cl_2_F_8_N_2_O_3_, the two aromatic rings are oriented at a dihedral angle of 50.12 (3)°. Intra­molecular N—H⋯O, C—H⋯O and N—H⋯Cl hydrogen bonds result in the formation of two six- and one five-membered rings. The six-membered rings have flattened-boat conformations, while the five-membered ring adopts an envelope conformation. In the crystal structure, inter­molecular N—H⋯O hydrogen bonds link the mol­ecules into centrosymmetric dimers.

## Related literature

For related literature, see: Drabek & Boeger (1986 ▶). For bond-length data, see: Allen *et al.* (1987 ▶). For ring conformation puckering parameters, see: Cremer & Pople (1975 ▶).
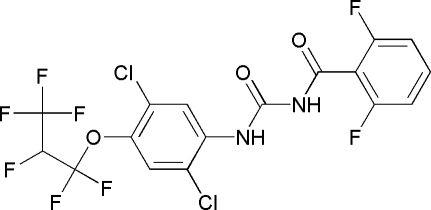


## Experimental

### 

#### Crystal data

C_17_H_8_Cl_2_F_8_N_2_O_3_
*M* *_r_* = 511.15
Monoclinic, 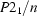

*a* = 9.2300 (18) Å
*b* = 16.404 (3) Å
*c* = 14.074 (3) Å
β = 108.77 (3)°
*V* = 2017.6 (8) Å^3^
*Z* = 4
Mo *K*α radiation
μ = 0.42 mm^−1^
*T* = 294 (2) K
0.40 × 0.30 × 0.20 mm

#### Data collection

Enraf–Nonius CAD-4 diffractometer
Absorption correction: ψ scan (North *et al.*, 1968 ▶) *T*_min_ = 0.851, *T*_max_ = 0.921
3609 measured reflections
3609 independent reflections
1922 reflections with *I* > 2σ(*I*)
3 standard reflections every 200 reflections intensity decay: none

#### Refinement

*R*[*F* ^2^ > 2σ(*F* ^2^)] = 0.086
*wR*(*F* ^2^) = 0.247
*S* = 1.09
3609 reflections
283 parameters
96 restraints
H-atom parameters constrained
Δρ_max_ = 0.42 e Å^−3^
Δρ_min_ = −0.34 e Å^−3^

### 

Data collection: *CAD-4 Software* (Enraf–Nonius, 1989 ▶); cell refinement: *CAD-4 Software*; data reduction: *XCAD4* (Harms & Wocadlo, 1995 ▶); program(s) used to solve structure: *SHELXS97* (Sheldrick, 2008 ▶); program(s) used to refine structure: *SHELXL97* (Sheldrick, 2008 ▶); molecular graphics: *ORTEP-3 for Windows* (Farrugia, 1997 ▶); software used to prepare material for publication: *SHELXTL* (Sheldrick, 2008 ▶).

## Supplementary Material

Crystal structure: contains datablocks I, global. DOI: 10.1107/S1600536808025506/hk2510sup1.cif

Structure factors: contains datablocks I. DOI: 10.1107/S1600536808025506/hk2510Isup2.hkl

Additional supplementary materials:  crystallographic information; 3D view; checkCIF report

## Figures and Tables

**Table 1:** Hydrogen-bond geometry (Å, °)

*D*—H⋯*A*	*D*—H	H⋯*A*	*D*⋯*A*	*D*—H⋯*A*
N2—H2*A*⋯Cl2	0.86	2.43	2.892 (4)	115
N2—H2*A*⋯O1	0.86	1.98	2.664 (6)	135
N1—H1*A*⋯O2^i^	0.86	1.98	2.814 (6)	163
C10—H10*A*⋯O2	0.93	2.28	2.851 (6)	119

Symmetry code: (i) 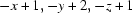.

**Fig. 1.**
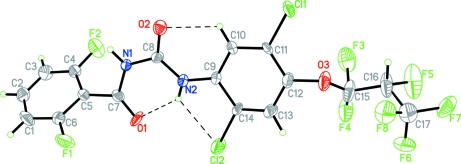
The molecular structure of the title compound, with the atom-numbering scheme. Displacement ellipsoids are drawn at the 50% probability level. Hydrogen bonds are shown as dashed lines.

**Fig. 2.**
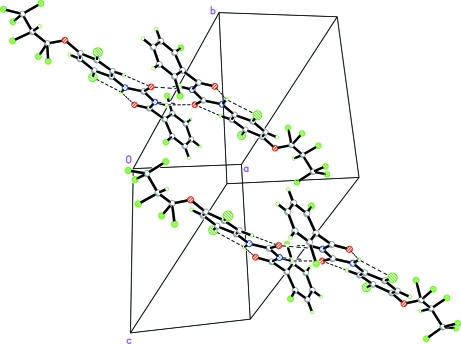
A packing diagram of the title compound. Hydrogen bonds are shown as dashed lines.

## supplementary crystallographic information

### Comment

The title compound is considered to belong to the fourth generation of insectides with properties such as high selectivity, low acute toxicity for mammals and high biological activity. It is generally recognized as a chitin-synthesis inhibitor that interrupts chitin-synthesis during the development and reproduction of the insectide. As part of our studies in this area, we report herein the crystal structure of the title compound.

In the molecule of the title compound, (Fig. 1) the bond lengths (Allen *et al.*,  1987) and angles are within normal ranges. Rings A (C1-C6) and B (C9-C14) are, of course, planar, and the dihedral angle between them is A/B = 50.12 (3)°. The intramolecular N-H···O, C-H···O and N-H···Cl hydrogen bonds (Table 1) result in the formation of two six- and one five-membered non-planar rings: C (O1/N1/N2/C7/C8/H2A), D (O2/N2/C8-C10/H10A) and E (Cl2/N2/C9/C14/H2A). Rings C and D adopt twisted [φ = -169.19 (2)°, θ = 21.09 (3)° (for ring C) and φ = 178.48 (3)°, θ = 127.74 (3)° (for ring D)] conformations, having total puckering amplitudes, Q_T_, of 0.113 (3) and 0.201 (3) Å, respectively (Cremer & Pople, 1975). Ring E adopts envelope conformation, with H2A atom displaced by 0.190 (3) Å from the plane of the other ring atoms.

In the crystal structure, intermolecular N-H···O hydrogen bonds (Table 1) link the molecules into centrosymmetric dimers (Fig. 2), in which they may be effective in the stabilization of the structure.

### Experimental

The title compound was prepared according to the literature method (Drabek & Boeger, 1986). The crystals suitable for X-ray analysis were obtained by dissolving the title compound (0.3 g) in acetonitrile (25 ml) and evaporating the solvent slowly at room temperature for about 8 d.

### Refinement

H atoms were positioned geometrically, with N-H = 0.86 Å (for NH) and C-H = 0.93 and 0.98 Å for aromatic and methine H, respectively, and constrained to ride on their parent atoms with U_iso_(H) = 1.2U_eq_(C,N).

### Crystal data

**Table:**

C_17_H_8_Cl_2_F_8_N_2_O_3_	*F*_000_ = 1016
*M**_r_* = 511.15	*D*_x_ = 1.683 Mg m^−^^3^
Monoclinic, *P*2_1_/*n*	Mo *K*α radiation λ = 0.71073 Å
Hall symbol: -P 2yn	Cell parameters from 25 reflections
*a* = 9.2300 (18) Å	θ = 9–12º
*b* = 16.404 (3) Å	µ = 0.42 mm^−^^1^
*c* = 14.074 (3) Å	*T* = 294 (2) K
β = 108.77 (3)º	Block, colorless
*V* = 2017.6 (8) Å^3^	0.40 × 0.30 × 0.20 mm
*Z* = 4	

### Data collection

**Table:**

Enraf–Nonius CAD-4 diffractometer	*R*_int_ = 0.0000
Radiation source: fine-focus sealed tube	θ_max_ = 25.2º
Monochromator: graphite	θ_min_ = 2.0º
*T* = 294(2) K	*h* = −11→10
ω/2θ scans	*k* = 0→19
Absorption correction: ψ scan(North *et al.*, 1968)	*l* = 0→16
*T*_min_ = 0.851, *T*_max_ = 0.921	3 standard reflections
3609 measured reflections	every 200 reflections
3609 independent reflections	intensity decay: none
1922 reflections with *I* > 2σ(*I*)	

### Refinement

**Table:**

Refinement on *F*^2^	Secondary atom site location: difference Fourier map
Least-squares matrix: full	Hydrogen site location: inferred from neighbouring sites
*R*[*F*^2^ > 2σ(*F*^2^)] = 0.086	H-atom parameters constrained
*wR*(*F*^2^) = 0.248	*w* = 1/[σ^2^(*F*_o_^2^) + (0.1258*P*)^2^ + 1.1819*P*] where *P* = (*F*_o_^2^ + 2*F*_c_^2^)/3
*S* = 1.09	(Δ/σ)_max_ = 0.001
3609 reflections	Δρ_max_ = 0.42 e Å^−^^3^
283 parameters	Δρ_min_ = −0.34 e Å^−^^3^
96 restraints	Extinction correction: none
Primary atom site location: structure-invariant direct methods	

### Special details

**Table:**

Geometry. All e.s.d.'s (except the e.s.d. in the dihedral angle between two l.s. planes) are estimated using the full covariance matrix. The cell e.s.d.'s are taken into account individually in the estimation of e.s.d.'s in distances, angles and torsion angles; correlations between e.s.d.'s in cell parameters are only used when they are defined by crystal symmetry. An approximate (isotropic) treatment of cell e.s.d.'s is used for estimating e.s.d.'s involving l.s. planes.
Refinement. Refinement of F^2^ against ALL reflections. The weighted R-factor wR and goodness of fit S are based on F^2^, conventional R-factors R are based on F, with F set to zero for negative F^2^. The threshold expression of F^2^ > 2sigma(F^2^) is used only for calculating R-factors(gt) etc. and is not relevant to the choice of reflections for refinement. R-factors based on F^2^ are statistically about twice as large as those based on F, and R- factors based on ALL data will be even larger.

### Fractional atomic coordinates and isotropic or equivalent isotropic displacement parameters (Å^2^)

**Table:**

	*x*	*y*	*z*	*U*_iso_*/*U*_eq_	
Cl1	1.21022 (15)	1.15534 (9)	0.61828 (12)	0.0527 (5)	
Cl2	1.06547 (19)	0.83402 (11)	0.80001 (17)	0.0914 (8)	
F1	0.4001 (5)	0.7889 (3)	0.7184 (3)	0.0975 (16)	
F2	0.4224 (4)	0.8023 (3)	0.3906 (3)	0.0810 (13)	
F3	1.5260 (5)	1.0359 (3)	0.6887 (5)	0.1116 (18)	
F4	1.5722 (4)	0.9618 (3)	0.8001 (4)	0.1039 (17)	
F5	1.8146 (6)	1.0579 (3)	0.7942 (5)	0.145 (3)	
F6	1.8127 (6)	1.0156 (3)	0.9703 (4)	0.1121 (18)	
F7	1.9281 (6)	1.1152 (3)	0.9350 (4)	0.119 (2)	
F8	1.6616 (6)	1.1347 (3)	0.9601 (4)	0.1029 (15)	
O1	0.6850 (4)	0.7921 (2)	0.6546 (4)	0.0703 (15)	
O2	0.6981 (4)	1.0216 (3)	0.5376 (3)	0.0505 (11)	
O3	1.4248 (4)	1.0694 (3)	0.7916 (4)	0.0758 (16)	
N1	0.5870 (4)	0.9045 (2)	0.5577 (3)	0.0359 (11)	
H1A	0.5033	0.9242	0.5175	0.043*	
N2	0.8483 (4)	0.9219 (2)	0.6377 (3)	0.0327 (10)	
H2A	0.8468	0.8724	0.6576	0.039*	
C1	0.1954 (8)	0.7296 (5)	0.5816 (6)	0.074 (2)	
H1B	0.1414	0.7140	0.6242	0.089*	
C2	0.1405 (7)	0.7183 (4)	0.4832 (5)	0.0588 (17)	
H2B	0.0444	0.6945	0.4569	0.071*	
C3	0.2142 (7)	0.7389 (4)	0.4199 (5)	0.0607 (17)	
H3A	0.1704	0.7275	0.3519	0.073*	
C4	0.3505 (6)	0.7758 (4)	0.4526 (4)	0.0450 (14)	
C5	0.4228 (6)	0.7893 (3)	0.5551 (4)	0.0435 (14)	
C6	0.3397 (7)	0.7666 (4)	0.6175 (5)	0.0568 (16)	
C7	0.5790 (6)	0.8247 (3)	0.5960 (4)	0.0437 (14)	
C8	0.7151 (6)	0.9534 (3)	0.5785 (4)	0.0407 (14)	
C9	0.9852 (5)	0.9614 (3)	0.6689 (4)	0.0330 (12)	
C10	1.0214 (5)	1.0332 (3)	0.6291 (4)	0.0368 (12)	
H10A	0.9477	1.0595	0.5770	0.044*	
C11	1.1661 (5)	1.0655 (3)	0.6667 (4)	0.0355 (12)	
C12	1.2807 (6)	1.0310 (4)	0.7494 (5)	0.0518 (16)	
C13	1.2445 (7)	0.9591 (4)	0.7901 (5)	0.0572 (17)	
H13A	1.3159	0.9350	0.8453	0.069*	
C14	1.1065 (5)	0.9252 (3)	0.7492 (4)	0.0391 (13)	
C15	1.5460 (7)	1.0386 (4)	0.8024 (7)	0.079 (3)	
C16	1.6854 (8)	1.0845 (5)	0.8271 (9)	0.107 (4)	
H16A	1.6576	1.1394	0.7999	0.129*	
C17	1.7772 (11)	1.0946 (6)	0.9319 (7)	0.089	

### Atomic displacement parameters (Å^2^)

**Table:**

	*U*^11^	*U*^22^	*U*^33^	*U*^12^	*U*^13^	*U*^23^
Cl1	0.0401 (8)	0.0436 (9)	0.0782 (11)	−0.0152 (6)	0.0242 (7)	−0.0007 (7)
Cl2	0.0513 (10)	0.0599 (12)	0.1330 (18)	−0.0100 (8)	−0.0123 (10)	0.0497 (12)
F1	0.097 (3)	0.135 (4)	0.051 (3)	−0.054 (3)	0.010 (2)	0.026 (2)
F2	0.086 (3)	0.103 (3)	0.059 (3)	−0.045 (3)	0.030 (2)	−0.034 (2)
F3	0.099 (4)	0.106 (4)	0.133 (5)	−0.034 (3)	0.041 (3)	−0.032 (3)
F4	0.054 (2)	0.072 (3)	0.172 (5)	−0.010 (2)	0.019 (3)	−0.015 (3)
F5	0.092 (3)	0.141 (5)	0.225 (7)	−0.023 (3)	0.082 (4)	−0.111 (5)
F6	0.127 (4)	0.104 (4)	0.095 (4)	−0.017 (3)	0.021 (3)	0.052 (3)
F7	0.099 (3)	0.083 (3)	0.116 (4)	−0.024 (3)	−0.048 (3)	0.006 (3)
F8	0.097 (3)	0.111 (4)	0.103 (4)	−0.001 (3)	0.034 (3)	−0.012 (3)
O1	0.029 (2)	0.047 (3)	0.107 (4)	0.0006 (18)	−0.017 (2)	0.037 (2)
O2	0.046 (2)	0.047 (3)	0.057 (3)	−0.0033 (19)	0.013 (2)	−0.005 (2)
O3	0.035 (2)	0.043 (3)	0.129 (5)	−0.009 (2)	−0.001 (2)	0.009 (3)
N1	0.028 (2)	0.019 (2)	0.058 (3)	0.0007 (17)	0.009 (2)	0.0093 (19)
N2	0.028 (2)	0.025 (2)	0.043 (3)	−0.0005 (17)	0.0085 (19)	−0.0044 (19)
C1	0.070 (4)	0.082 (5)	0.076 (4)	−0.028 (4)	0.032 (4)	0.017 (4)
C2	0.046 (3)	0.051 (4)	0.071 (4)	−0.016 (3)	0.007 (3)	0.003 (3)
C3	0.059 (4)	0.061 (4)	0.051 (4)	−0.023 (3)	0.002 (3)	−0.014 (3)
C4	0.045 (3)	0.046 (3)	0.042 (3)	−0.003 (3)	0.011 (3)	−0.004 (3)
C5	0.039 (3)	0.042 (3)	0.047 (3)	−0.006 (2)	0.011 (2)	0.010 (3)
C6	0.044 (3)	0.067 (4)	0.053 (4)	−0.009 (3)	0.007 (3)	0.014 (3)
C7	0.045 (3)	0.039 (3)	0.047 (4)	−0.010 (3)	0.016 (3)	0.004 (3)
C8	0.037 (3)	0.027 (3)	0.051 (4)	0.000 (2)	0.005 (3)	0.009 (3)
C9	0.026 (2)	0.036 (3)	0.036 (3)	0.000 (2)	0.009 (2)	−0.005 (2)
C10	0.032 (2)	0.040 (3)	0.042 (3)	−0.007 (2)	0.016 (2)	−0.003 (2)
C11	0.033 (3)	0.035 (3)	0.044 (3)	−0.009 (2)	0.020 (2)	0.008 (2)
C12	0.030 (3)	0.053 (4)	0.065 (4)	−0.003 (2)	0.006 (3)	0.004 (3)
C13	0.042 (3)	0.051 (4)	0.071 (4)	0.013 (3)	0.007 (3)	0.017 (3)
C14	0.022 (2)	0.042 (3)	0.049 (3)	0.012 (2)	0.005 (2)	0.009 (2)
C15	0.029 (3)	0.050 (5)	0.142 (8)	0.006 (3)	0.007 (4)	−0.014 (4)
C16	0.041 (4)	0.046 (4)	0.219 (11)	−0.009 (3)	0.021 (5)	0.007 (6)
C17	0.080	0.089	0.096	−0.006	0.026	0.004

### Geometric parameters (Å, °)

**Table:**

Cl1—C11	1.727 (5)	C2—C3	1.326 (9)
Cl2—C14	1.751 (6)	C2—H2B	0.9300
F1—C6	1.398 (7)	O3—C15	1.191 (7)
F2—C4	1.327 (7)	O3—C12	1.418 (6)
F3—C15	1.552 (10)	C3—C4	1.338 (8)
F4—C15	1.284 (8)	C3—H3A	0.9300
F5—C16	1.477 (9)	C4—C5	1.399 (8)
F6—C17	1.403 (10)	C5—C6	1.389 (8)
F7—C17	1.420 (10)	C5—C7	1.488 (7)
F8—C17	1.415 (10)	C9—C10	1.390 (7)
O1—C7	1.186 (6)	C9—C14	1.439 (7)
N1—C8	1.380 (6)	C10—C11	1.374 (7)
N1—C7	1.427 (7)	C10—H10A	0.9300
N1—H1A	0.8600	C11—C12	1.414 (7)
C1—C2	1.327 (10)	C12—C13	1.398 (8)
C1—C6	1.401 (9)	C13—C14	1.338 (8)
C1—H1B	0.9300	C13—H13A	0.9300
N2—C8	1.347 (6)	C15—C16	1.434 (9)
N2—C9	1.361 (6)	C16—C17	1.455 (13)
N2—H2A	0.8600	C16—H16A	0.9800
O2—C8	1.245 (6)		
			
C8—N1—C7	126.7 (4)	C11—C10—C9	120.1 (5)
C8—N1—H1A	116.6	C11—C10—H10A	120.0
C7—N1—H1A	116.6	C9—C10—H10A	120.0
C2—C1—C6	115.8 (6)	C10—C11—C12	122.7 (5)
C2—C1—H1B	122.1	C10—C11—Cl1	120.0 (4)
C6—C1—H1B	122.1	C12—C11—Cl1	117.2 (4)
C8—N2—C9	125.8 (4)	C13—C12—C11	117.4 (5)
C8—N2—H2A	117.1	C13—C12—O3	121.2 (5)
C9—N2—H2A	117.1	C11—C12—O3	121.4 (5)
C1—C2—C3	124.0 (6)	C14—C13—C12	119.7 (5)
C1—C2—H2B	118.0	C14—C13—H13A	120.2
C3—C2—H2B	118.0	C12—C13—H13A	120.2
C15—O3—C12	125.5 (6)	C13—C14—C9	123.9 (5)
C2—C3—C4	120.9 (6)	C13—C14—Cl2	118.8 (4)
C2—C3—H3A	119.6	C9—C14—Cl2	117.2 (4)
C4—C3—H3A	119.6	O3—C15—F4	126.1 (6)
F2—C4—C3	122.4 (6)	O3—C15—C16	122.7 (7)
F2—C4—C5	117.0 (5)	F4—C15—C16	111.1 (6)
C3—C4—C5	120.7 (6)	O3—C15—F3	94.9 (6)
C6—C5—C4	115.6 (5)	F4—C15—F3	84.7 (6)
C6—C5—C7	121.6 (5)	C16—C15—F3	93.7 (7)
C4—C5—C7	122.8 (5)	C15—C16—C17	119.4 (9)
C5—C6—F1	116.8 (5)	C15—C16—F5	121.4 (7)
C5—C6—C1	122.9 (6)	C17—C16—F5	94.9 (6)
F1—C6—C1	120.1 (6)	C15—C16—H16A	106.6
O1—C7—N1	123.1 (5)	C17—C16—H16A	106.6
O1—C7—C5	125.0 (5)	F5—C16—H16A	106.6
N1—C7—C5	111.9 (5)	F6—C17—F8	115.2 (7)
O2—C8—N2	125.8 (5)	F6—C17—F7	95.9 (6)
O2—C8—N1	116.9 (4)	F8—C17—F7	134.6 (8)
N2—C8—N1	117.2 (5)	F6—C17—C16	105.8 (7)
N2—C9—C10	126.3 (5)	F8—C17—C16	95.3 (7)
N2—C9—C14	117.6 (5)	F7—C17—C16	107.7 (7)
C10—C9—C14	116.0 (4)		

### Hydrogen-bond geometry (Å, °)

**Table:**

*D*—H···*A*	*D*—H	H···*A*	*D*···*A*	*D*—H···*A*
N2—H2A···Cl2	0.86	2.43	2.892 (4)	115
N2—H2A···O1	0.86	1.98	2.664 (6)	135
N1—H1A···O2^i^	0.86	1.98	2.814 (6)	163
C10—H10A···O2	0.93	2.28	2.851 (6)	119

Symmetry codes: (i) −*x*+1, −*y*+2, −*z*+1.
